# A novel, small-volume subcutaneous furosemide formulation delivered by an abdominal patch infusor device in patients with heart failure: results of two phase I studies

**DOI:** 10.1093/ehjcvp/pvad073

**Published:** 2023-10-06

**Authors:** Joanna Osmanska, Katriona Brooksbank, Kieran F Docherty, Stacy Robertson, Kirsty Wetherall, Alex McConnachie, Jerry Hu, Roy S Gardner, Andrew L Clark, Iain B Squire, Paul R Kalra, Pardeep S Jhund, Pieter Muntendam, John J V McMurray, Mark C Petrie, Ross T Campbell

**Affiliations:** School of Cardiovascular and Metabolic Health, British Heart Foundation Glasgow Cardiovascular Research Centre, University of Glasgow, Glasgow G12 8TA, UK; Department of Cardiology, Queen Elizabeth University Hospital, Glasgow G51 4TF, UK; School of Cardiovascular and Metabolic Health, British Heart Foundation Glasgow Cardiovascular Research Centre, University of Glasgow, Glasgow G12 8TA, UK; School of Cardiovascular and Metabolic Health, British Heart Foundation Glasgow Cardiovascular Research Centre, University of Glasgow, Glasgow G12 8TA, UK; Department of Cardiology, Queen Elizabeth University Hospital, Glasgow G51 4TF, UK; School of Cardiovascular and Metabolic Health, British Heart Foundation Glasgow Cardiovascular Research Centre, University of Glasgow, Glasgow G12 8TA, UK; Robertson Centre for Biostatistics, University of Glasgow G12 8TB, UK; Robertson Centre for Biostatistics, University of Glasgow G12 8TB, UK; Allucent, Durham, NC 27713, USA; Scottish National Advanced Heart Failure Service, Golden Jubilee National Hospital, Glasgow G81 4DY, UK; Department of Academic Cardiology, Hull University Teaching Hospital NHS Trust, Hull GU16 5JQ, UK; Department of Cardiovascular Sciences, University of Leicester, and NIHR Cardiovascular Biomedical Research Centre, Glenfield Hospital, Leicester LE3 9QP, UK; Department of Cardiology, Queen Alexandra Hospital, Portsmouth Hospitals University NHS Trust, Portsmouth PO6 3LY, UK; School of Cardiovascular and Metabolic Health, British Heart Foundation Glasgow Cardiovascular Research Centre, University of Glasgow, Glasgow G12 8TA, UK; SQ Innovation Inc., Burlington, MA 01803, USA; School of Cardiovascular and Metabolic Health, British Heart Foundation Glasgow Cardiovascular Research Centre, University of Glasgow, Glasgow G12 8TA, UK; School of Cardiovascular and Metabolic Health, British Heart Foundation Glasgow Cardiovascular Research Centre, University of Glasgow, Glasgow G12 8TA, UK; Department of Cardiology, Glasgow Royal Infirmary, Glasgow G40SF, UK; School of Cardiovascular and Metabolic Health, British Heart Foundation Glasgow Cardiovascular Research Centre, University of Glasgow, Glasgow G12 8TA, UK; Department of Cardiology, Queen Elizabeth University Hospital, Glasgow G51 4TF, UK

**Keywords:** Heart failure, Furosemide, Subcutaneous, Pharmacokinetics, Pharmacodynamics, Intravenous

## Abstract

**Aims:**

Subcutaneous (SC) furosemide has potential advantages over intravenous (IV) furosemide by enabling self-administration or administration by a lay caregiver, such as facilitating early discharge, preventing hospitalizations, and in palliative care. A high-concentration, pH-neutral furosemide formulation has been developed for SC administration via a small patch infusor pump. We aimed to compare the bioavailability, pharmacokinetic (PK), and pharmacodynamic (PD) profiles of a new SC furosemide formulation with conventional IV furosemide and describe the first use of a bespoke mini-pump to administer this formulation.

**Methods and results:**

A novel pH-neutral formulation of SC furosemide containing 80 mg furosemide in ∼2.7 mL (infused over 5 h) was investigated. The first study was a PK/PD study of SC furosemide compared with 80 mg IV furosemide administered as a bolus in ambulatory patients with heart failure (HF). The primary outcome was absolute bioavailability of SC compared with IV furosemide. The second study investigated the same SC furosemide preparation delivered by a patch infusor in patients hospitalized with HF. Primary outcome measures were treatment-emergent adverse events, infusion site pain, device performance, and PK measurements.

The absolute bioavailability of SC furosemide in comparison to IV furosemide was 112%, resulting in equivalent diuresis and natriuresis. When SC furosemide was administered via the patch pump, there were no treatment-emergent adverse events and 95% of participants reported no/minor discomfort at the infusion site.

**Conclusion:**

The novel preparation of SC furosemide had similar bioavailability to IV furosemide. Administration via a patch pump was feasible and well tolerated.

## Graphical Abstract

**Graphical Abstract:**
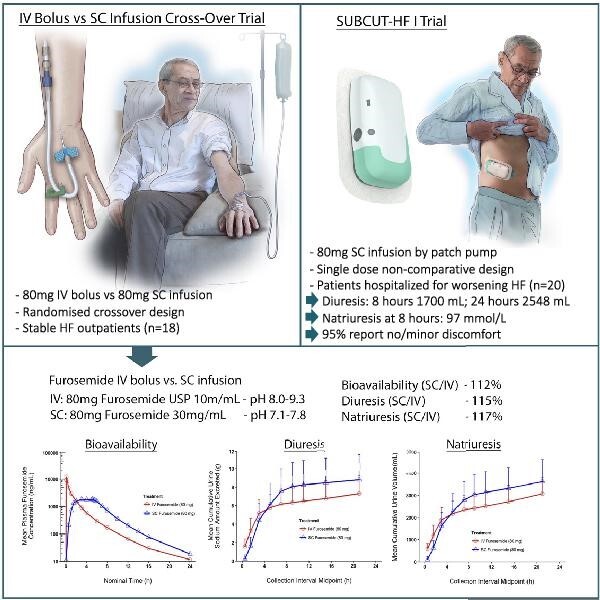

## Introduction

The ability to deliver furosemide subcutaneously (SC) rather than intravenously (IV) may result in opportunities to achieve a diuresis in clinical scenarios where parenteral administration is desirable but IV administration is unattractive or impractical. Prior attempts to deliver furosemide SC have involved large volumes as the concentration of conventional furosemide (10 mg/mL) means that 80 mg, for example, of furosemide requires an 8-mL syringe. Conventional infusion pumps used for SC infusion are often large and bulky, which makes them impractical to use except for in-patients confined to hospital beds. Conventional furosemide for injection is alkaline^1^ and has low bioavailability when administered SC.^2,3^ These limitations have resulted in the use of SC furosemide being restricted to small numbers of patients in niche clinical settings. Therefore, a concentrated, small-volume, pH-neutral, furosemide formulation that can be administered SC with similar bioavailability to IV furosemide, delivered by a small, bespoke pump, would be attractive. This paper reports two ‘first-in-human’ studies using a novel furosemide preparation specifically designed for SC administration. This novel formulation of SC furosemide (SQIN-Furosemide) has two key attributes. First, it is pH neutral, so it is less irritant to the skin. Second, it is concentrated with 80 mg SC furosemide delivered in a volume of 2.7 mL. Because of these properties, we hypothesized (a) that SC delivery of novel SC furosemide would be well tolerated and would achieve similar bioavailability and a diuresis equivalent to IV administration and (b) that SC furosemide could be delivered by a small pump applied to the skin of the abdomen and, potentially, worn under clothing. The first study was a pharmacokinetic (PK) and pharmacodynamic (PD) study of the novel SC furosemide compared with IV furosemide delivered by a large pump in ambulatory patients with heart failure (HF) (SQIN-Furosemide PK/PD, NCT04384653). In the second study, the same novel preparation of SC furosemide was delivered by a small abdominal pump in patients hospitalized due to HF (SQIN-Furosemide/abdominal device, NCT04846816).

## Methods

A complete description of the methods for both trials is provided in the Supplementary Appendix. A summary of the methods for both trials is provided below.

### Study 1—phase I PK/PD study of SC furosemide (SQIN-Furosemide PK/PD, NCT04384653)

**Figure 1:**
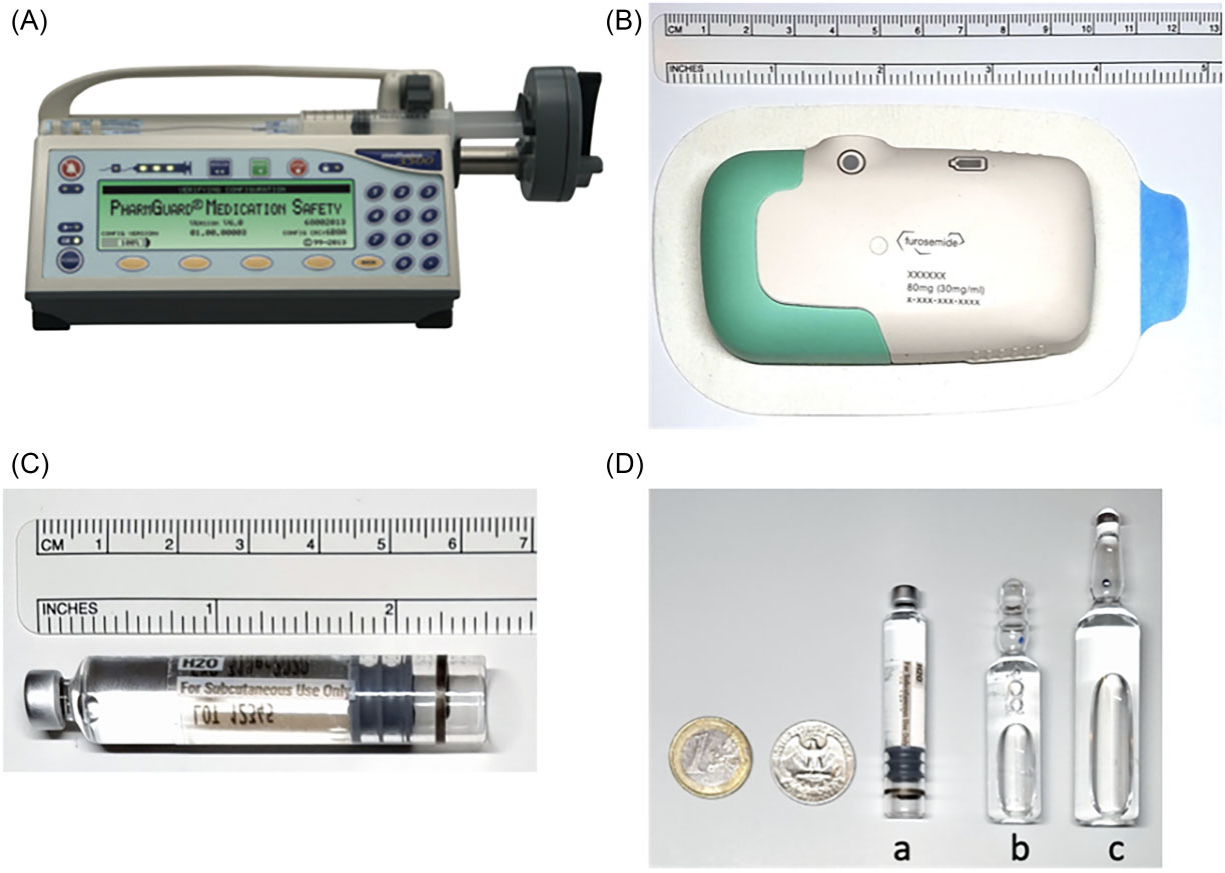
Infusion devices and SC furosemide vial used to administer SC furosemide in SQIN-Furosemide PK/PD and SQIN-Furosemide/abdominal device studies. (*A*) Medfusion 3500 (v6) precision infusion pump (Smiths Medical ASD Inc., Minneapolis, MN, USA); (*B*) SQIN-Infusor (SQ Innovation Inc., Burlington, MA, USA); (*C*) pH-neutral SC furosemide, 80 mg in 2.7 mL; and (*D*) size of (a) SC furosemide vial in comparison to (b) 5 mL 50 mg IV furosemide vial and (c) 10 mL IV 100 mg IV furosemide vial.

*Drug*: The investigational SC furosemide formulation (SC SQIN-Furosemide) was a Captisol (Ligand Pharmaceuticals Incorporated, Emeryville, CA, USA) buffered solution with a concentration of 30 mg/mL at pH 7.4 (range: 7.0–7.8). Each vial contained 80 mg furosemide in ∼2.7 mL (*Figure 1*C and D), which was administered by SC infusion over 5 h using a biphasic delivery profile of 30 mg over the first hour and 12.5 mg/h for the next 4 h. The comparator was an FDA (Food and Drug Administration)-approved IV furosemide formulation (Hospira, Inc., Lake Forest, IL, USA), administered as an 80 mg IV bolus over 2 min (furosemide 10 mg/mL in solution at alkaline pH of 8.0–9.3).

*Infusion device*: FDA-approved Medfusion 3500 (v6) precision infusion pump (Smiths Medical ASD Inc., Minneapolis, MN, USA; *Figure 1*A).

*Study design*: PK/PD of SC furosemide compared with IV furosemide in an open-label, single-dose, randomized, active-comparator, crossover single-centre study in 20 adults with chronic HF.

*Patients*: Patients had chronic HF, New York Heart Association (NYHA) class II or III, treated with oral furosemide at a dose of ≥40 mg per day and estimated glomerular filtration rate (eGFR) ≥45 mL/min per 1.73 m^2^. A full description of inclusion and exclusion criteria is provided in the Supplementary Appendix. Study activity took place at a single site, DeLand Clinical Research Unit in DeLand, FL, USA. The study was approved by the Institutional Review Board. All patients provided written consent and the trial was done in accordance with the Declaration of Helsinki and Good Clinical Practice guidelines.

**Figure 2:**
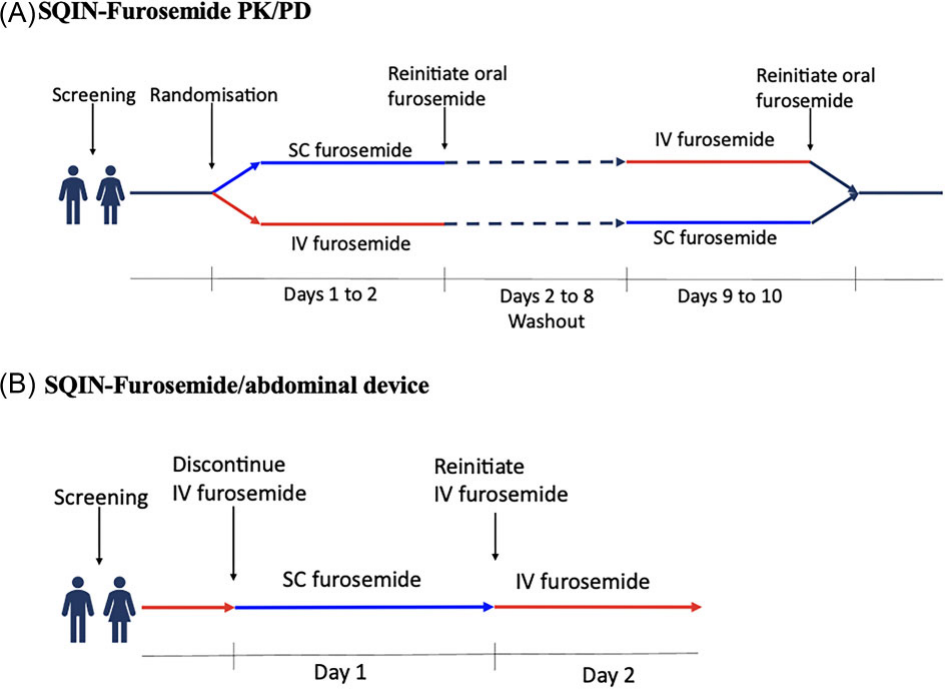
Study design of (*A*) SQIN-Furosemide PK/PD and (*B*) SQIN-Furosemide/abdominal device studies.

*Randomization and masking*: Patients were randomly assigned 1:1 in a crossover design to receive a single dose of open-label 80 mg furosemide administered as an IV bolus over 2 min (treatment A) or 80 mg SC furosemide administered SC over 5 h using a Medfusion 3500 (v6) precision infusion pump (treatment B). Patients were randomized to receive the study drugs in sequence AB (IV followed by SC) or BA (SC followed by IV) with a 7-day washout period in between treatments (*Figure 2*A). The study was open label, with both patients and investigators aware of treatment assignment.

*Outcome measures*: The primary outcome was relative absolute bioavailability following a 5-h SC infusion based on a comparison of the area under the curve (AUC) of SC furosemide and IV furosemide. Secondary outcomes included measures of PK and PD parameters of both SC furosemide and IV furosemide, and any infusion site pain and skin reactions. A more detailed description of the secondary outcomes assessed is provided in the Supplementary Appendix.

*Statistical analyses*: The analysis population included all subjects with sufficient concentration–time data to calculate the PK profile for at least one treatment. The PK population consisted of all subjects who received at least one dose of the study drug and had at least one furosemide PK concentration. The safety population consisted of all subjects who received at least one dose of the study drug. The relative absolute bioavailability of SC furosemide in comparison to IV furosemide was calculated using the following equation: (area under the curve from time 0 to infinity [AUCinf] SC furosemide/dose of SC furosemide)/(AUCinf IV furosemide/dose of IV furosemide). Derived plasma PK descriptive statistics were tabulated by dosing group and summary statistics were generated. The PD variables (urine volume and total urine sodium concentration) were assessed using a linear repeated measures mixed-effect model appropriate for a two-period crossover design with treatment and period as fixed effects. A heterogeneous-compound symmetry covariance matrix was used to allow for unequal treatment variances and to model the correlation between the two treatment measurements within each subject. The Kenward–Roger method was used to calculate the denominator degrees of freedom for the fixed effects. The geometric least-squares mean (GLSM) difference between the treatment groups, 90% confidence interval (CI), and *P*-value were calculated. Statistical analysis was performed using Phoenix WinNolin version 8.2 or later (Certara, Princeton, NJ, USA), R Software versions 3.6 and 3.4.0 (R Foundation for Statistical Computing, Vienna, Austria), R studio version 1.0.143 (R Foundation for Statistical Computing, Vienna, Austria), and SAS version 9.4 (SAS, Cary, NC, USA).

### Study 2—phase II study of SC SQIN-Furosemide and abdominal device (SQIN-Infusor) combination (SQIN-Furosemide/abdominal device, NCT04846816)

*Drug*: The same investigational SC furosemide formulation (SC SQIN-Furosemide) was used in Study 2 as in Study 1. As in Study 1, the SC infusion of SC furosemide was performed using a biphasic delivery profile of 30 mg of SC furosemide over the first hour, followed by 50 mg for 4 h to deliver 80 mg (∼2.7 mL) of the SC furosemide formulation over 5 h.

*Infusion device*: A novel abdominal patch infusor device (SQIN-Infusor, SQ Innovation Inc., Burlington, MA, USA, *Figure 1*B). This device is a bespoke system, adapted from the design of a SC insulin pump. The SQIN-Infusor was attached to the abdominal skin of participants using an adhesive patch made of a 3M 1529 adhesive tape (3M, St. Paul, MN, USA). The device places a 29G needle in the SC tissue at the start of delivery and withdraws the needle upon completion of drug administration. The dimensions of the device are 9.3 cm by 5.0 cm by 2.2 cm.

*Study design*: The SQIN-Furosemide/abdominal device study was a prospective, single-centre, open-label, single-arm, single-dose study of SC furosemide administered by the SQIN-Infusor (*Figure 2*B).

*Patients*: Patients being treated in hospital for a primary diagnosis of HF (any ejection fraction [EF]) requiring ongoing treatment with IV furosemide at a dose of ≥40 mg/day, and eGFR ≥30 mL/min per 1.73 m^2^. Full inclusion/exclusion criteria are provided in the Supplementary Appendix. Patients were enrolled in a single site, at the Queen Elizabeth University Hospital, Glasgow, United Kingdom. The study was approved by the Yorkshire & The Humber—Leeds West Research Ethics Committee, UK. All patients provided written consent and the trial was done in accordance with the Declaration of Helsinki and Good Clinical Practice guidelines.

*Outcome measures*: The primary outcomes included adverse events, infusion site pain (assessed using a 10-point numeric rating scale with 0 indicating ‘no pain’ and 10 indicating ‘the most intense pain imaginable’), device failure (failure of the device to administer study drug and adhesion of the SQIN-Infusor using a five-point scale), and PK (plasma furosemide concentration was measured at 0 [pre-dose], 60, and 240 min after the start of SC furosemide infusion). Secondary outcomes included PD parameters (urine volume and spot urine sodium concentration at 8 h), assessment of skin irritation (using a six-point scale), and patient acceptability (using the System Usability Scale [SUS], consisting of 10 questions with five-point response options from 1 [strongly disagree] to 5 [strongly agree]. The scale provides a score from 0 to 100, with scores >85 representing exceptional usability and a score <70 representing unacceptable usability.^4^ A detailed description of the study outcomes is provided in the Supplementary Appendix.

*Statistical analysis:* The analysis population included all participants in whom the SQIN-Infusor was activated. All primary and secondary safety outcomes were listed by participant or summarized using descriptive statistics, as appropriate. All statistical analyses were performed using the SAS version 9.4 software package (SAS Institute Inc., Cary, NC, USA).

## Results

### Study 1—phase I PK/PD study of SC SQIN-Furosemide conducted (SQIN-Furosemide PK/PD, NCT04384653)

#### Patients

A total of 20 volunteers with ambulatory NYHA II/III HF (no EF inclusion criterion) were enrolled between 8 October 2020 and 11 June 2021. Two participants did not receive the full dose of SC SQIN-Furosemide (due to inadequate line priming of the infusion pump [Medfusion 3500 (v6)]) and were excluded. All remaining participants (*n* = 18) completed both cross-over treatments (*Table 1*). The median age of the participants was 71 years (IQR [interquartile range] 64–74 years) and 13 (72%) were male. A total of 16 (89%) were NYHA II and 2 (11%) were NYHA III. All participants were diagnosed with HF at least 12 months before enrolment. All participants were treated with oral furosemide before the study, with a median daily dose of 40 mg (IQR 40–80 mg).

**Table 1:** Baseline characteristics of patients in phase I SQIN-Furosemide PK/PD study and phase I study of SQIN-Furosemide/abdominal device combination

	SQIN-Furosemide PK/PD study (*n* = 18)	SQIN-Furosemide/abdominal device trial (*n* = 20)
Age, years	71 [64–74]	75 [64–85]
Male	13 (72%)	11 (55%)
BMI, kg/m^2^	32 [28–36]	30 [26–35]
NYHA class		
II	16 (89%)	5 (25%)
III	2 (11%)	14 (70%)
IV	0	1 (5%)
Heart rate, beats/min	69 [63–79]	75 [63–88]
Systolic blood pressure, mmHg	131 [119–136]	127 [109–143]
Jugular venous distention	NR	15 (75%)
Rales/diminished breath sounds	NR	20 (100%)
Orthopnoea	NR	16 (80%)
Paroxysmal nocturnal dyspnoea	NR	12 (60%)
LVEF %	NR	36 (30–50)
HF duration, *n* (%)		
<6 months	0	15 (75%)
6–12 months	0	1 (5%)
>12 months	18 (100%)	4 (20%)
Aetiology of HF, *n* (%)		
Ischaemic	NR	4 (20%)
Non-ischaemic or other	NR	16 (80%)
Comorbidities, *n* (%)		
HTN	18 (100%)	11 (55%)
PCI/CABG	7 (39%)	2 (10%)
Diabetes mellitus	6 (33%)	7 (35%)
Anaemia	3 (17%)	14 (70%)
Atrial fibrillation	4 (22%)	11 (55%)
CRT, *n* (%)	4 (22%)	1 (5%)
HF medication, *n* (%)		
ACEi/ARB/ARNi	16 (89%)	13 (65%)
Beta-blocker	13 (72%)	12 (60%)
MRA	4 (22%)	7 (35%)
SGLT2i	0	4 (20%)
Digoxin	2 (11%)	8 (40%)
Blood tests		
NT-proBNP, pg/mL	NR	5184 [1922–6488]
Haemoglobin, g/dL	136 [127–145]	124 [117–130]
Sodium, mmol/L	140 [139–143]	138 [137–142]
Potassium, mmol/L	4.2 [3.8–4.7]	4.1 [3.9–4.3]
eGFR, mL/min per 1.73 m^2^	68 [60–79]	45 [41–59]

Continuous data presented as median (interquartile range [IQR]).

ACEi, angiotensin-converting enzyme inhibitor; ARB, angiotensin receptor blocker; ARNi, angiotensin receptor–neprilysin inhibitor; BMI, body mass index; CABG, coronary artery graft bypass; CRT, cardiac resynchronization therapy; eGFR, estimated glomerular filtration rate; HF, heart failure; HTN, hypertension; LVEF, left ventricular ejection fraction; MRA, mineralocorticoid receptor antagonist; NR, not recorded; NT-proBNP, N-Terminal pro-B-type natriuretic peptide; NYHA, New York Heart Association; PCI, percutaneous coronary intervention; and SGLT2i- sodium–glucose co-transporter-2 inhibitor.

#### Primary outcome

The relative absolute bioavailability of SC SQIN-Furosemide in comparison to IV furosemide was 112% (90% CI: 104, 120%).

##### Secondary outcomes

###### Pharmacokinetics

**Figure 3:**
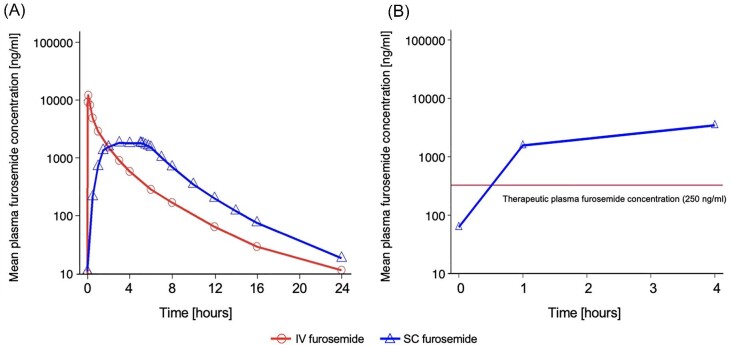
Mean plasma furosemide concentration (*A*) following administration of SC furosemide and IV furosemide in SQIN-Furosemide PK/PD study and (*B*) following administration of SC furosemide delivered by SQIN-Infusor.

Plasma concentrations of furosemide were higher with IV furosemide than SC furosemide for the first 2 h but following this SC furosemide plasma furosemide concentrations were consistently higher than IV furosemide (*Figure 3*A). The GLSM of maximum plasma concentrations (*C*_max_) was 2060 ng/mL and 13 600 ng/mL with SC furosemide and IV furosemide, respectively (*Table 2*). Median *C*_max_ was

1940 ng/mL for SC furosemide and 14 400 ng/mL for IV furosemide. Median time to *C*_max_ (*T*_max_) was 5 and 0.08 h for SC furosemide and IV furosemide, respectively. Median AUC from time 0 to the last measurable plasma concentration (AUClast) was 13 600 and 11 600 h*ng/mL with SC furosemide and IV furosemide, respectively. Median AUC from time 0 to infinity (AUCinf) was 13 700 and 11 700 h*ng/mL for SC furosemide and IV furosemide, respectively. Median half-life (t^½^) was 3.70 h for SC furosemide and 3.55 h for IV furosemide. Median apparent systemic clearance for SC furosemide was 5820 mL/h, median systemic clearance for IV furosemide was 6870 mL/h. Median volume of distribution was 30 500 and 34 400 L for SC furosemide and IV furosemide, respectively (*Table 3*).

**Table 2:** Pharmacokinetic and pharmacodynamic results for SC furosemide and IV furosemide in the SQIN-Furosemide PK/PD study

	SC SQIN-Furosemide	IV furosemide	Treatment difference SC vs. IV (%) (90% CI)	*P-*value
Pharmacokinetic outcomes				
*C*_max_ (GLSM) (ng/mL)	2060	13 600	15.12 (13.67, 16.72)	<0.0001
*T*_max_ (median) (h)	5.00	0.08	NC	NC
AUClast (GLSM) (h*ng/mL)	13 300	11 900	111.61 (103.68, 120.15)	0.018
AUCinf (GLSM) (h*ng/mL)	13 400	12 000	111.92 (103.98, 120.46)	0.015
Pharmacodynamic outcomes				
Urine volume 8 h (GLSM) (mL)	2664.3	2284.6	116.6 (99.5, 136.8)	0.078
Urine volume 24 h (GLSM) (mL)	3501.4	3020.0	115.9 (100.2, 134.2)	0.065
Urinary sodium excretion 8 h (GLSM) (g)	7.1	6.0	118.4 (102.5, 136.7)	0.033

AUCinf, plasma concentration to infinity; AUClast, last measurable plasma concentration; *C*_max_, the peak plasma concentration; IV, intravenous; GLSM, geometric least-squares mean; NC, not calculated; SC, subcutaneous; and *T*_max_, the time from time 0 (pre-dose) to the peak plasma concentration.

**Table 3:** Pharmacokinetic results of the SQIN-Furosemide PK/PD study

	*C*_max_ (ng/mL)	*T*_max_ (h)	AUC_last_ (h*ng/mL)	AUC_0-24_ (h*ng/mL)	AUC_inf_ (h*ng/mL)	*t*_1/2_ (h)	*Vz*/*F* (L)	CL (mL/h)
SC furosemide 5-h infusion								
*n*	18	18	18	18	18	18	18	18
Mean	2010	NC	13 000	13 000	13 100	3.71	34 200	6360
SD	391	NC	2510	2510	2550	0.68	11 600	1520
CV%	19.5	NC	19.3	19.3	19.4	18.4	34.0	23.8
Geometric mean	1970	NC	12 800	12 800	12 900	3.65	32 700	6220
Geometric CV%	20.7	NC	21.6	21.6	21.7	17.7	29.7	21.7
Min	1210	2.0	7800	7800	7850	2.58	21 300	4650
Median	1940	5.0	13 600	13 600	13 700	3.70	30 500	5820
Max	2690	5.75	17 000	17 000	17 200	5.57	68 200	10 200
IV furosemide bolus administration								
*n*	18	18	18	18	18	18	18	18
Mean	13 800	NC	11 900	12 000	12 000	3.67	37 900	7180
SD	4100	NC	3380	3370	3400	1.25	19 900	2070
CV%	29.8	NC	28.3	28.2	28.3	34.2	52.5	28.9
Geometric mean	13 100	NC	11 500	11 500	11 600	3.47	34 600	6920
Geometric CV%	34.8	NC	28.6	28.4	28.5	35.6	42.4	28.5
Min	6180	0.03	6450	6480	6480	1.91	21 200	3770
Median	14 400	0.08	11 600	11 600	11 700	3.55	34 400	6870
Max	21 800	0.25	21 000	21 000	21 200	6.70	107 000	12 300

AUC, area under the curve; AUC_inf_, plasma concentration to infinity; AUC_last_, last measurable plasma concentration; CL, systemic clearance; *C*_max_, the peak plasma concentration; CV, coefficient of variation; *t*_1/2_, terminal phase elimination half-life; *T*_max_, the time from time 0 (pre-dose) to the peak plasma concentration; and *Vz*/*F*, volume of distribution.

One IV furosemide subject was found to have an unexplained excessively high concentration of furosemide at 2 and 5 min; these two results were excluded from the analysis.

###### Pharmacodynamics

####### Urine output.

**Figure 4:**
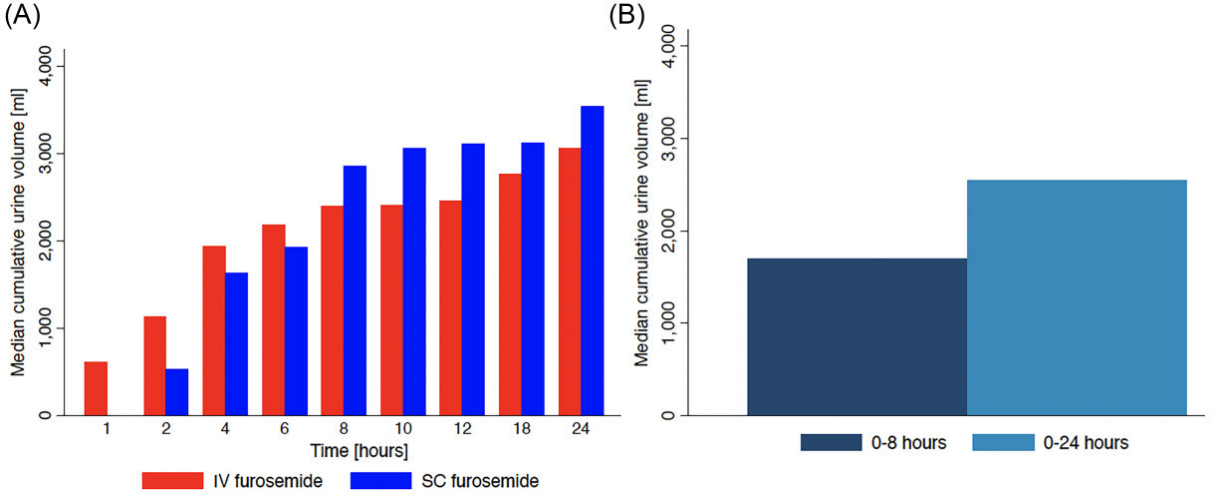
Urine volumes following (*A*) administration of SC furosemide and IV furosemide in SQIN-Furosemide PK/PD study and (*B*) SC furosemide delivered by SQIN-Infusor.

**Figure 5:**
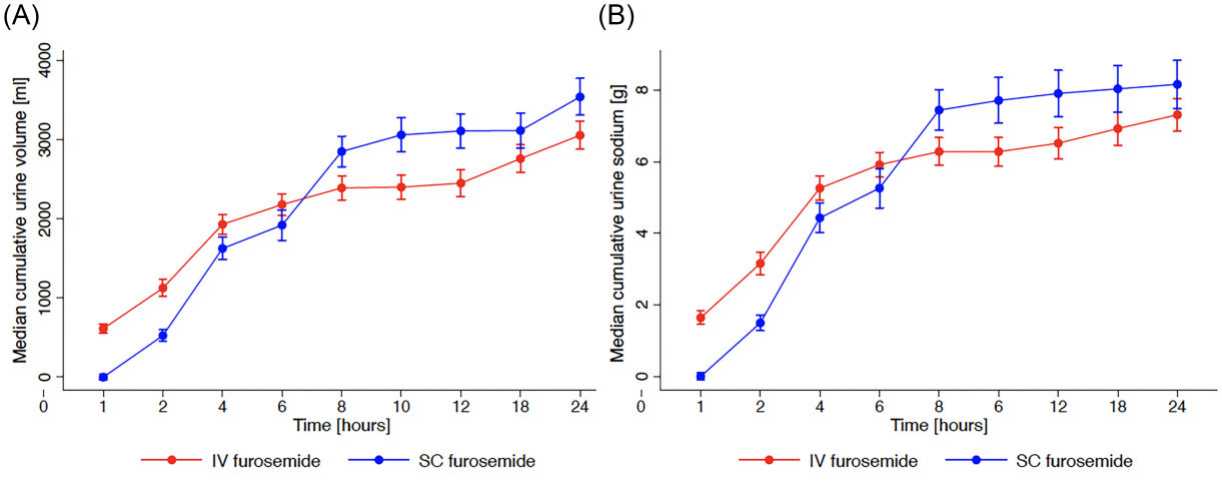
Cumulative (*A*) urine volumes (median and standard error) and (*B*) sodium excretion (median and standard error) following administration of SC furosemide and IV furosemide in the SQIN-Furosemide PK/PD study.

At 8 and 24 h, there was no difference between the GLSM of urine output achieved with SC furosemide vs. IV furosemide (8 h—2664 mL vs. 2285 mL, treatment difference 117% [90%. CI: 99.6—137], *P*-value 0.078; 24 h—3501 mL vs. 3020 mL treatment difference 116% [90% CI: 100–134], *P*-value 0.065) (*Table 2* and *Figure 4*A). Treatment with SC furosemide was associated with slower onset and more gradual diuresis than IV furosemide (*Figure 5*A).

####### Total urinary sodium concentration.

Treatment with SC furosemide resulted in higher GLSM of urine sodium excretion than IV furosemide at 8 h—7.1 g for SC furosemide vs. 6.0 g, treatment difference 118% (95% CI: 103–137), *P*-value 0.033 (*Table 2* and *Figure 5*B).

###### Infusion site pain and skin reactions

Five (28%) participants reported pain or discomfort during treatment with SC furosemide. One participant reported pain of 6 (scale 1–10), with the remaining participants reporting a pain score between 0 and 4. The median score was 0 (range 0–6). Participants reported pain at the time of removal and placement of the infusion set (*n* = 3 for both). Local skin reactions were noted in four (22%) participants with SC furosemide. The maximum score was 1 (well-defined erythema), observed in one participant at the time of removal of the infusion set.

#### Adverse events

There were four adverse events (AEs) reported (Supplementary Table S4). Two AEs were attributed to study treatment: (1) maximum pain at the infusion site reported as 6 out of 10 (subsequently improved to 2 out of 10) and (2) an episode of orthostatic hypotension resulting in early discontinuation of SC infusion (10 min before the planned end of infusion). All AEs resolved with no long-term sequelae.

### Study 2—phase I study of SC SQIN-Furosemide and abdominal device combination (NCT04846816)

#### Patients

A total of 20 patients hospitalized with HF, requiring treatment with IV furosemide, were enrolled between 6 May 2021 and 13 August 2021 (*Table 1*). The median age of the participants was 75 years (IQR 64–85 years) and 11 (55%) were male. The median EF was 36% (IQR 30–50%). A total of 12 (60%) had HF with reduced EF (HFrEF i.e. LVEF ≤ 40%) and 8 (40%) had HF with preserved EF (HFpEF i.e. LVEF > 40%). A total of 15 out of 20 (75%) had been diagnosed with HF in the preceding 6 months. Overall, nine (45%) participants had received treatment with oral diuretics before admission to the hospital, with a median dose of 40 mg per day (IQR 20–40 mg). The median daily dose of IV furosemide was 100 mg (IQR 50–100 mg) at the time of enrolment.

#### Primary outcome measures

There were no treatment-related adverse events.

*Infusion site pain.* Treatment with SC furosemide administered with SQIN-Infusor was well tolerated by the participants. Of 12 (60%) who reported injection site discomfort during treatment, 8 reported this to be a discomfort only at the time of needle insertion. The maximal pain score was 5 (scale 0–10), reported by one participant.

*Device failure.* In one participant, 1 h and 25 min from the start of the infusion the dressing became loose and the SQIN-Infusor detached from the participant's skin. The individual was overweight (BMI [body mass index] 40 kg/m^2^) and was sweating on a hot day. In the other 19 patients, there were no device malfunctions; the full contents of the vial were delivered.

*Pharmacokinetics.* All subjects (*n* = 20, 100%) achieved plasma furosemide level ≥250 ng/mL at 60 min with a median concentration of 1155 ng/mL (IQR 848–1665 ng/mL). Plasma furosemide levels ≥250 ng/mL at 240 min were achieved by all participants who had furosemide levels measured at this time (*n* = 19, one participant was excluded due to early discontinuation of treatment due to malfunctioning adhesive) with median plasma furosemide concentration of 2730 ng/mL (IQR 2460–3380 ng/mL) (*Figure 3*B).

#### Secondary outcome measures

*Urine output.* Urine volumes over 8 h (secondary outcome) and 24 h (exploratory analysis) are depicted in *Figure 4*B. The median urine output at 8 h was 1700 mL (IQR 1215–2600 mL). The median urine output 24 h from the start of SC furosemide administration was 2548 mL (median IQR 2025–3570 mL).

*Urinary sodium concentration.* The median spot urine sodium concentration at 8 h was 97 mmol/l (IQR 85–112 mmol/L).

*Local skin reactions.* A local skin reaction occurred in 4 participants, which, in all cases, was a transient, faint, indistinct erythema (score 0.5).

*Patient acceptability.* The median SUS score was 99 (IQR 84–100) with 14 (70%) participants scoring above 85 (excellent usability).

## Discussion

In these two first-in-human studies, we compared the bioavailability, PK, and PD profiles of a new SC furosemide formulation with conventional furosemide injected IV by bolus in ambulatory patients with HF and the first use of a bespoke mini-pump (SQIN-Infusor) used to administer this formulation in hospitalized patients with HF. The bioavailability of SC furosemide delivered by a conventional pump was very similar to that of IV furosemide. As expected, conventional furosemide administered as an IV bolus resulted in a more rapid rise in plasma furosemide concentration than an infusion of SC furosemide, which resulted in a slower rise and longer plateau in plasma concentrations. SC furosemide resulted in similar diuresis to IV furosemide boluses. Similar plasma furosemide concentrations and diuresis were achieved when SC furosemide was delivered both through both a traditional and a bespoke abdominal SC infusion device.

The abdominal patch pump (SQIN-Infusor) is a modified insulin pump, so it has an established record of ease of use. In keeping with this, the SQIN-Infusor was well tolerated by all patients with few reports of discomfort or pain on needle injection. One of the 20 pumps became loose when the adhesive detached from a patient during the infusion. A balance must be struck between using adhesives that when too sticky can cause difficulty during pump removal and adhesives that may become detached if not sufficiently adhesive. In clinical practice, if a device detaches, there is the option of applying a further device if necessary. Use of the device is straightforward; there is single-button activation and light and sound signals to indicate the status of the device and the progress of treatment. The administration rate of SC furosemide by SQIN-Infusor cannot be changed by the user, which minimizes the risk of drug administration errors. The pump can be worn under a patient's clothing and allows mobilization.

Prior experience of furosemide administered SC comes primarily from a small number of non-randomized studies that included small numbers of patients. These reports used off-licence SC administration of conventional furosemide.^5,6^ The PK and PD parameters of furosemide administered SC were not described in these studies, and furosemide administered SC was associated with localized skin reactions in nearly one in every four patients.^5,7–9^ There has been one other furosemide formulation developed for SC administration (scPharmaceuticals, Burlington, MA, USA).^10^ This pH-neutral SC furosemide is not as concentrated, containing 8 mg furosemide/mL (compared with 30 mg/mL in the current studies). Compared to IV furosemide, this other SC preparation delivered by a conventional infusion pump had a similar diuretic effect when compared with IV furosemide in a randomized trial of 40 patients with HF.^11^

This formulation also reported complete bioavailability at 100%, whereas relative bioavailability in our trial was 112%. There are notable differences in the trial design that explain the higher bioavailability (and >100%) in our trial. Specifically, in our trial, the comparator was a one-off bolus of 80 mg of IV furosemide, where the comparator in the trial reported by Sica *et al*.^10^ was two 40 mg IV boluses administered 2 h apart, which may have resulted in different plasma levels of furosemide in the comparator arms (i.e. higher in the two boluses of 40 mg). The split bolus reflects the first study of 41 HF patients treated with IV furosemide^12^ and FDA prescribing information that recommends an initial dose of up to 40 mg with a repeat dose after 2 h.^13^ We considered a split bolus regimen, but chose a single dose of 80 mg instead as this is more commonly used in routine clinical care in our experience. This resulted in markedly higher *C*_max_ (2060 ng/mL in our trial vs. 1990 ng/mL reported by Sica *et al.*^10^). As furosemide is renally excreted, these high initial concentrations are excreted without contributing proportionally to the diuretic effect. As a result of the concentration-dependent elimination of the high peak concentration, the AUC is lower than with a slower infusion and accompanied by a slight loss in diuresis when compared with the slower infusion. In fact, this observation of our controlled cross-over study with measurements of plasma furosemide supports the hypothesis of the Diuretic Strategies in Patients with Acute Decompensated Heart Failure (DOSE) trial,^14^ although this did not translate into clinical endpoints in this large randomized clinical trial, potentially attributable to the very high variability in diuretic response.

We envisage that the use of SC furosemide delivered by this small pump will involve both the patient and caregiver. Education around SC furosemide delivery will inform both the patient and caregiver about the functions of the device and will include detailed explanations of the goals and practicalities of decongestion. The potential value of this approach is to provide decongestion at home, either to avoid admission to hospital or to expedite discharge from hospital and reduce the length of stay. Ambulatory care for HF is a key priority for many healthcare systems around the world, given the potential saving of bed days and cost.^15^ Managing patients outside the hospital became even more important during the COVID-19 pandemic. In the United Kingdom, a randomized controlled trial is comparing early discharge of patients hospitalized with HF enabled by the SQIN-Furosemide/SQIN-Infusor combination with ‘usual care’ in-hospital decongestion (NCT 05419115). This trial will assess the safety and efficacy of this drug/device combination.

### Limitations

We compared an SC infusion of furosemide with an IV bolus rather than an SC infusion with an IV infusion. A comparison of an infusion of SC furosemide vs. an infusion of IV furosemide might have produced different results. IV boluses are, however, the most common route of parenteral furosemide administration in clinical practice and no difference in fluid balance and weight change between bolus and infusion was found in the DOSE trial.^14^ The SQIN-Furosemide PK/PD study used a conventional SC pump to administer the SC furosemide. Due to technical issues with the priming of the conventional infusion pump, the treatment was not reliably administered in two participants who were subsequently excluded from our analysis. This reflects the real-life challenges of the use of conventional SC pumps.

The first study was conducted in a small number (*n* = 18) of patients with stable HF requiring treatment with oral diuretics, who may not be representative of all patients with HF requiring treatment with parenteral diuretics. When SC furosemide was delivered by the abdominal pump, the pump was applied by an investigator. This does not reflect the intended use of SQIN-Furosemide (i.e. primarily by the patient and carer in the home).

## Conclusion

The novel formulation SC furosemide had similar bioavailability and resulted in similar diuresis and higher natriuresis in comparison to conventional IV furosemide delivered as a bolus. This novel SC furosemide preparation can be safely and effectively administered by a bespoke mini abdominal pump.

## Supplementary Material

pvad073_Supplemental_File

## Data Availability

The data underlying this article will be shared on reasonable request to the corresponding author.

## Notes

**Conflict of interest:** J.O., K.B., S.R., K.W., A.M., and A.L.C. have no disclosures relevant to the contents of this paper. K.F.D. has received research grant support from Boehringer Ingelheim and speaker's fees from AstraZeneca. R.S.G. is an investigator for Biotronik, Boston Scientific, Lumira, and Medtronic and consultant for Abbott, AstraZeneca, Boehringer Ingelheim, Boston Scientific, Novartis, Pharmacosmos, and Roche Diagnostics. I.B.S. has received research grant support from Novartis and fees for participation in advisory boards and educational events from Novartis, AstraZeneca, and Vifor. P.R.K. has received research grant support from British Heart Foundation, Pharmacosmos, and Vifor and consultancy fees or honoraria from Ackea, Amgem, AstraZeneca, Bayer, Boehringer Ingelheim, Boston Scientific, Napp, Novartis, Pharmacosmos, Servier, and Vifor. P.S.J. has received research funding from Boehringer Ingelheim and Analog Devices Inc. and speaker's fees from AstraZeneca and Novartis. P.S.J. is a member of Advisory Board at AstraZeneca, Boehringer Ingelheim, and Novartis and has been remunerated for their time working on the DAPA-HF and DELIVER by AstraZeneca, the PARADIGM-HF and PARAGON-HF trials by Novartis, SOUL by Novo Nordisk, and FINEARTS-HF by Bayer. P.M. is a shareholder and employee of SQ Innovation Inc. J.J.V.M. has received payments through Glasgow University for work on clinical trials, and for consulting and other activities from Alnylam, Amgen, AstraZeneca, Bayer, Boehringer Ingelheim, BMS, Cardurion, Cytokinetics, Dal-Cor, GSK, Ionis, KBP Biosciences, Novartis, Pfizer, and Theracos. J.J.V.M. has received personal lecture fees from the Corpus, Abbott, Hikma, Sun Pharmaceuticals, Medscape/Heart.Org, Radcliffe Cardiology, and Servier and is Director of Global Clinical Trial Partners (GCTP). M.P. has received research funding from Boehringer Ingelheim, Roche, SQ Innovation Inc., AstraZeneca, Novartis, Novo Nordisk, Medtronic, Boston Scientific, and Pharmacosmos. M.P. is a member of Consultancy and Trial committees at Boehringer Ingelheim, Novartis, AstraZeneca, Novo Nordisk, Abbvie, Bayer, Takeda, Corvia, Cardiorentis, Pharmacosmos, Siemens, and Vifor and is Director of GCTP. M.P. and J.J.M. are supported by a British Heart Foundation Centre of Research Excellence Grant RE/18/6/34217. R.T.C. has received consultancy fees from Boehringer Ingelheim.
