# Supplementary material for: A novel, small-volume subcutaneous furosemide formulation delivered by an abdominal patch infusor device in patients with heart failure: results of two phase I studies
Source: Eur Heart J Cardiovasc Pharmacother. 2023 Oct 6;10(1):35–44. doi: 10.1093/ehjcvp/pvad073 (PMC10766906; doi:10.1093/ehjcvp/pvad073)
Supplement: pvad073_Supplemental_File [file pvad073_supplemental_file.docx]

**Supplementary Materials**

[**Supplementary Table 1. Inclusion and exclusion criteria for SQIN-Furosemide PK/PD trial. 2**](#_Toc144139257)

[**Supplementary Table 2. Inclusion and exclusion criteria for SQIN- Furosemide/abdominal device trial. 6**](#_Toc144139258)

[**Supplementary Table 3. Primary and secondary outcomes for SQIN-Furosemide PK/PD and SQIN-Furosemide/abdominal device trials. 8**](#_Toc144139259)

**Supplementary Table 4. Summary of adverse events from both trials ............................10**

**Supplementary Methods........................................................................................................11**

[**Study 1 - Phase 1 PK/PD study of SC SQIN-Furosemide conducted in Florida, USA (SQIN-Furosemide PK/PD, NCT04384653) 11**](#_Toc144139261)

[**Study 2 - Phase 1 study of SC SQIN-Furosemide and abdominal device (SQIN-Infusor) combination in the United Kingdom (SQIN-Furosemide/abdominal device, NCT04846816) 16**](#_Toc144139262)

**Supplementary Table 1. Inclusion and exclusion criteria for SQIN-Furosemide PK/PD trial.**

| **Inclusion Criteria** | **Exclusion Criteria** |
| --- | --- |
| An Institutional Review Board (IRB) approved informed consent is signed and dated prior to any study-related activities. | Acute Decompensated Heart Failure (ADHF) or recent history of hospitalisation for heart failure in the last 4 weeks |
| Male and female subjects ≥18 and ≤ 80 years of age, with body weight <130 kg and body mass index (BMI) <38 kg/m2. | Worsening of signs or symptoms of heart failure in the two weeks prior to the Screening, or those expected to require IV loop diuretics or inpatient treatment for heart failure during the study |
| Females will be non-pregnant, non-lactating, or post-menopausal, or surgically sterile (e.g., tubal ligation, hysterectomy). | Systolic blood pressure (SBP) <90 mmHg |
| Females of childbearing potential will use TWO of the following forms of contraception: intrauterine device (IUD), IUD with spermicide, female condom with spermicide, contraceptive sponge with spermicide, an intravaginal system, diaphragm with spermicide, cervical cap with spermicide, a male sexual partner who agrees to use a male condom with spermicide, a sterile sexual partner. | Temperature ≥38°C (oral or equivalent) or sepsis or active infection requiring IV anti-microbial treatment |
| History of at least 3 months treated heart failure (NYHA class II/III) with presence of symptoms of chronic volume overload requiring ongoing treatment with oral furosemide at a dose of ≥40 mg per day for at least 30 days prior to Day -1 | Serum sodium <130 mmol/l and serum potassium <3.5 mmol/l |
| Agrees to abstain from using alcohol, caffeine-containing products, and tobacco-/nicotine-containing products while in residence at the Clinical Research Unit (CRU) | Significant other cardiac abnormalities which may interfere with study participation or study assessments. |
| Able to participate in the study in the opinion of the Investigator | Current or planned treatment during the study with any IV therapies, including inotropic agents, vasopressors, levosimendan, nesiritide or analogues; or mechanical support (intra-aortic balloon pump, endotracheal intubation, mechanical ventilation, or any ventricular assist device) |
| Has the ability to understand the requirements of the study and is willing to comply with all study procedures | Subject is cachectic |
|  | Diagnosed with Type I diabetes mellitus or Type II diabetes requiring insulin therapy |
|  | Presence or need for urinary catheterisation, urinary tract abnormality, or disorder interfering with urination |
|  | Impaired renal function, defined as an estimated glomerular filtration rate (eGFR) on admission <45 ml/min/1.73m2, calculated using the simplified Modification of Diet in Renal Disease (sMDRD) equation |
|  | Indication of moderate-to-severe hepatic dysfunctions as determined by the Investigator |
|  | Administration of IV radiographic contrast agent within 72 hours prior to Screening or acute contrast-induced nephropathy at the time of Screening |
|  | Major surgery within 30 days prior to Screening |
|  | Administration of an investigational drug or implantation of investigational device, or participation in another interventional trial, within 30 days prior to Screening |
|  | Any surgical or medical condition, which in the opinion of the Investigator may pose an undue risk to the subject, interfere with participation in the study, or which may affect the integrity of the study data |
|  | Positive test for hepatitis B (HBsAg), hepatitis C (HCV), or human immunodeficiency virus (HIV) at Screening |
|  | Any positive urine drug screen at Screening or clinic admission |
|  | Concomitant use of any drugs known to interact with furosemide |
|  | History of alcohol abuse within 6 months prior to Screening and/or signs or symptoms of alcoholism, as determined by the Investigator |
|  | Any positive alcohol test on admission to the CRU |
|  | History of severe allergic or hypersensitivity reactions to furosemide |
|  | Donation of greater than 100 ml of either whole blood or plasma within 30 days prior to study drug administration |
|  | Been informed of possible COVID-19 exposure in past 4 weeks, or recent onset of signs or symptoms of possible COVID-19 infection, including cough, shortness of breath, or temperature ≥ 38°C |
|  | Travelled via airplane or cruise ship within the last 14 days |

**Supplementary Table 2. Inclusion and exclusion criteria for SQIN- Furosemide/abdominal device trial.**

| **Inclusion Criteria** | **Exclusion Criteria** |
| --- | --- |
| Written informed consent | Unable to consent to inclusion in study due to lack of capacity |
| Male or female ≥18 years of age | Requiring treatment with intravenous furosemide at dose >200 mg per day as determined by the usual care team |
| Meet European Society of Cardiology (ESC) criteria for diagnosis of HF | Current inotropes, vasopressors or intra-aortic balloon pump therapy |
| Inpatient with a primary diagnosis of HF requiring treatment with intravenous furosemide at dose | Concomitant use of diuretics in 12 hours preceding administration of study drug with SQIN-Infusor |
|  | Systolic blood pressure (SBP) <90 mmHg |
|  | Pregnancy or breastfeeding |
|  | Left sided valve disease with planned surgery or percutaneous intervention |
|  | Type 1 myocardial infarction during index hospitalisation (type 2 myocardial infarctions are allowed) |
|  | Any surgical or medical condition which prevents patient from ambulation during the infusion |
|  | Renal impairment, defined as eGFR < 30 ml/min/1.73 m2 at screening |
|  | Patient on active cardiac transplant waiting list |
|  | Potassium <3.0 mmol/l |
|  | Potassium >6.0 mmol/l |
|  | Sodium <125 mmol/l |
|  | Any contraindications for furosemide administration as per furosemide SmPC |
|  | Any surgical or medical conditions, which in the opinion of the investigator may pose an undue risk to the subject, interfere with participation in the study or which may affect the integrity of the data |

**Supplementary Table 3. Primary and secondary outcomes for SQIN-Furosemide PK/PD and SQIN-Furosemide/abdominal device trials.**

| **SQIN-Furosemide PK/PD** | **SQIN-Furosemide/abdominal device** |
| --- | --- |
| **PRIMARY OUTCOMES** | |
| Pharmacokinetics (relative absolute bioavailability of 5 hours SC infusion of SQIN-Furosemide in comparison to IV furosemide bolus) | Safety as determined by treatment emergent adverse events (including serious adverse events) and adverse drug events (including serious adverse drug events) |
|  | Infusion site pain |
|  | Any device failures |
|  | Pharmacokinetics (plasma furosemide concentration at 0, 60 and 240 minutes from start of infusion) |
| **SECONDARY OUTCOMES** | |
| Pharmacokinetics (including maximum plasma concentration (Cmax), time to Cmax (Tmax), area under the concentration versus time curve (AUC) from time 0 (pre-dose) to 24 hours post dose (AUC 0-24), AUC from time 0 to the last measurable plasma concentration (AUClast) and to infinity (AUCinf), half-life (t½), apparent systemic clearance and volume of distribution (SC only), and systemic clearance and volume of distribution (IV only) | Pharmacodynamics (urine volume and spot urine sodium concentration at 8 hours from start of infusion) |
| Pharmacodynamics (urine volume and total urine sodium concentration at 8 and 24 hours post dose) | Local skin reactions |
| Infusion site pain and local skin reactions | Patient acceptability |

IV- intravenous; SC- subcutaneous;

**Supplementary Table 4. Summary of adverse events from both trials.**

| **Adverse Event** | **Severity** | **Related to study treatment** | **Serious Adverse Event (SAE)** |
| --- | --- | --- | --- |
| **SQIN-Furosemide PK/PD study** | | | |
| Headache | Mild | Not related | No |
| Upper respiratory tract infection | Mild | Not related | No |
| Orthostatic hypotension | Moderate | Related | No |
| Infusion site pain | Moderate | Related | No |
| **SQIN-Furosemide/abdominal device trial** | | | |
| Flare up of gout | Mild | Possibly | No |
| Transient hypotension | Mild | Not related | No |
| Fatigue | Mild | Not related | No |

**Supplementary Methods**

**Study 1 - Phase 1 PK/PD study of SC SQIN-Furosemide conducted in Florida, USA (SQIN-Furosemide PK/PD, NCT04384653)**

**Drug:** The investigational SC furosemide formulation (SQIN-Furosemide) was a Captisol^®^ (Ligand Pharmaceuticals Incorporated, Emerville, CA, USA) buffered solution with a concentration of 30 mg/ml at pH 7.4 (range: 7.0 to 7.8). Each vial contained 80 mg furosemide in ~2.7ml. 80 mg furosemide was administered by SC infusion over 5 hours using a biphasic delivery profile of 30 mg in first hour and 12.5 mg/hour for 4 hours. The comparator IV furosemide formulation (Hospira, Inc., Lake Forest, IL, USA), was administered as an IV bolus over 2 minutes. It contains furosemide 10 mg/ml in solution at alkaline pH of 8.0 to 9.3.

**Infusion device:** a Medfusion 3500 (v6) precision infusion pump (Smiths Medical ASD Inc, Minneapolis, MN, USA - Figure 1A).

**Study design:** PK/PD of SQIN-Furosemide compared with IV furosemide in an open-label, single-dose, randomised, active-comparator, crossover single centre study in 20 adults with chronic heart failure (HF).

**Patients**: Patients with chronic HF, New York Heart Association (NYHA) class II or III, between 18 and 80 years of age with body weight <130 kg and body mass index (BMI) <38kg/m^2^ were eligible for inclusion if they had at least a 3-month history of treatment for HF. Participants were required to have symptoms requiring ongoing treatment with oral furosemide, at a dose of ≥40 mg per day, for at least 30 days prior to randomisation. Key exclusion criteria included acute decompensated HF or a recent (4 weeks) history of hospitalisation for HF; worsening of signs or symptoms of HF in the 2 weeks prior to screening; systolic blood pressure (SBP) <90 mmHg. A full list of inclusion and exclusion criteria are detailed in the Supplementary Table 1. All patients were recruited at DeLand Clinical Research Unit in DeLand, FL, USA.

**Randomisation and study drug**

Patients were randomly assigned 1:1 in a crossover design to receive a single dose of open-label 80 mg furosemide (10 mg/ml) administered as IV bolus over 2 min (Treatment A) or 80 mg SC furosemide (30 mg/ml) administered SC over 5 hours using a Medfusion 3500 (v6) precision infusion pump (Treatment B). Patients were randomised to receive the study drugs in sequence AB (IV followed by SC) or BA (SC followed by IV) with a 7-day washout period in between treatments (Figure 2A).

**Study procedures**

Oral furosemide was withheld prior to administration of study drug for at least 24 hours. Venous blood samples for pharmacokinetic (PK) analysis were collected during IV and SC infusions at pre-specified time points, selected as recommended in FDA and European Medicines Agency (EMA) guidelines.^18^

**IV furosemide:** 0 (pre-dose), 2 minutes (immediately after the IV bolus injection was complete), 5, 15, 30, 60, 120, and 180 minutes, and at 4, 6, 8, 12, 16, and 24 hours after the start of infusion.

**SC furosemide:** 0 (pre-dose) and at 30, 60, 90, 120, 180, 240, 300 (immediately after completion of the infusion), 305, 315, 330, and 345 minutes, and at 6, 7, 8, 10, 12, 14, 16, and 24 hours after the start of infusion.

Urine was collected over 24 hours, with measurement of urinary volume made at pre-specified time points (8 and 24 hours after start of the infusion).

After a 7-day washout period all participants received the alternative study drug following the protocol described above.

**Outcome measures**

*Primary outcome:*

The primary outcome was relative absolute bioavailability following 5-hour SC infusion based on a comparison of area under curve (AUC) SC furosemide: IV furosemide.

*Secondary outcomes:*

*Pharmacokinetics:* PK parameters over the timeframe of 24 hours, including, but not limited to maximum plasma concentration (Cmax), time to Cmax (Tmax), AUC versus time curve from time 0 (pre-dose) to 24 hours post dose (AUC 0-24), AUC from time 0 to the last measurable plasma concentration (AUClast) and to infinity (AUCinf), half-life (t½), apparent systemic clearance and volume of distribution (SC only), and systemic clearance and volume of distribution (IV only)

*Pharmacodynamics:* Urine volume and sodium concentration in urine collected over 8 hours and 24 hours post-dose.

*Infusion site pain and skin reactions* (SC furosemide only)*:* Infusion site pain was assessed using a 10-point numerical rating scale (NRS) with 0 indicating “no pain” and 10 indicating “the most intense pain imaginable” at the time of placement of the infusion set, maximal pain during infusion and at the time of removal. Assessment for skin reactions was completed with photography and using a standardised scale to assess erythema and oedema formation as recommended by the National Academy of Sciences. This 6-point scale ranges from 0 representing “no response” to 4 representing “bullous (large blister), spreading, or other severe reaction”.^19^ Skin appearance was assessed pre-dosing, upon removal of SC infusion set, 6 and 24 hours after start of the infusion.

**Blood sample analysis**

Blood plasma samples were collected at prespecified nominal timepoints, processed and stored at -20^o^ C or colder until assayed using liquid chromatography tandem-mass spectrometry analytical method (range 5 to 5000 ng/L calibration range).

**Statistical analyses**

*Analysis Populations*

The analysis population included all subjects with sufficient concentration-time data to calculate the PK profile for at least one treatment and without a protocol deviation that affected the PK profile. The PK population consisted of all subjects who received at least 1 dose of study drug and had at least one furosemide PK concentration. The safety population consisted of all subjects who received at least 1 dose of study drug.

*Bioavailability Analysis*

Relative absolute bioavailability of SC furosemide was calculated using the following equation: (AUCinf SC furosemide/ dose of SC furosemide) / (AUCinf IV furosemide/dose of IV furosemide).

*PK Analysis*

Furosemide concentrations were summarised using descriptive statistics for each treatment. Derived plasma PK descriptive statistics were tabulated by dosing group and summary statistics. Descriptive statistics for PK parameters include the arithmetic mean (all parameters) and geometric mean (for Cmax, AUClast, and AUCinf, only), coefficient of variation (CV), standard deviation (SD) of the arithmetic mean, median, minimum, maximum, and number). PK parameters (Cmax, AUClast and AUCinf) of the analysis population were assessed using a linear repeated measures mixed-effect model appropriate for a 2-period crossover design with treatment and period as fixed effects. A heterogeneous-compound symmetry covariance matrix was used to allow for unequal treatment variances and to model the correlation between the 2 treatment measurements within each subject. The Kenward-Roger method was used to calculate the denominator degrees of freedom for the fixed effects. A log transformation was applied to the Cmax, AUClast and AUCinf data. Ninety percent (90%) confidence intervals (CIs), based on the t-distribution, were generated from the above mixed-effect model for the geometric least square mean (GLSM) ratios for Cmax, AUClast and AUCinf to compare SC to IV treatment.

*Pharmacodynamic Analysis*

PD variables (urine volume and total urine sodium concentration) were assessed using a linear repeated measures mixed-effect model appropriate for a 2-period crossover design with treatment and period as fixed effects. A heterogeneous-compound symmetry covariance matrix was used to allow for unequal treatment variances and to model the correlation between the 2 treatment measurements within each subject. The Kenward-Roger method was used to calculate the denominator degrees of freedom for the fixed effects. The GLSM difference between the treatment groups, 90% CIs and p-value were calculated.

*Safety Analysis*

All safety data were listed by subject.

**Study 2 - Phase 1 study of SC SQIN-Furosemide and abdominal device (SQIN-Infusor) combination in the United Kingdom (SQIN-Furosemide/abdominal device, NCT04846816)**

**Drug:** The same investigational furosemide formulation (SQIN-Furosemide) was used in Study 2 as in Study 1. As in Study 1, the SC infusion of SC furosemide was performed using a biphasic delivery profile of 30 mg of SC furosemide over 60 minutes, followed by 50 mg for 4 hours to deliver 80 mg (~2.7 ml) of the SC furosemide formulation over 5 hours.

**Infusion device**: A novel abdominal patch infusor device (SQIN-Infusor, SQ Innovation Inc, Burlington, MA, USA, Figure 1B). This device is a bespoke system, adapted from the design of a SC insulin pump. The SQIN-Infusor was attached to the abdominal skin of participants using an adhesive patch made of a 3M 1529 adhesive tape (3M, St Paul, MN, USA). The device places a 29G needle in the SC tissue at the start of delivery and withdraws the needle upon completion of drug administration. The dimensions of the device are 9.3 cm by 5.0 cm by 2.2 cm.

**Study design:** The SQIN-Furosemide/abdominal device study was a prospective, single-center, open-label, single-arm, single-dose study designed to investigate the safety and tolerability of SC furosemide administered by the SQIN-Infusor.

**Patients:** Patients were eligible for inclusion if they were hospitalised with a primary diagnosis of HF (any ejection fraction) requiring ongoing treatment with IV furosemide at a dose of ≥40 mg/day. Key exclusion criteria included treatment with IV furosemide at a dose of >200mg/day, SBP <90 mmHg and an estimated glomerular filtration rate (eGFR) <30 ml/min/1.73m^2^). A full list of inclusion and exclusion criteria are detailed in the Supplementary Table 2. All patients were recruited at the Queen Elizabeth University Hospital, Glasgow, Scotland, United Kingdom.

**Study procedures:** IV furosemide was withheld prior to administration of study drug for at least 12 hours. Patients were prescribed a single dose of SC furosemide 80 mg which was administered over 5 hours by the SQIN-Infusor. SQIN-Infusor was applied by a member of trial team and patients were asked to continue with all in-patient activities as usual during the infusion. Blood samples to measure plasma furosemide concentration were collected at 0 (pre-dose), 60 and 240 min after start of infusion. Urinary volume and urine spot sodium concentration were measured at 8 hours.

**Outcome measures**

*Primary outcomes:*

1. *Safety:* Safety was assessed in the 24-hours following initiation of study drug infusion by recording treatment emergent adverse events (TEAEs [including serious adverse events (SAEs)]) and adverse drug events (ADEs [including serious adverse drug events (SADEs)]).
2. *Infusion site pain*: Infusion site pain was assessed using a 10-point NRS with 0 indicating “no pain” and 10 indicating “the most intense pain imaginable”. Assessments were made at the start of treatment, at the point of maximal pain during SC furosemide infusion, at the time of device removal and at 24 hours after the start of the infusion.
3. *Device failure:* Device failure to administer study drug was recorded by the research team. Adhesion of the SQIN-Infusor device was assessed in accordance with the FDA draft Guidance Assessing Adhesion with Transdermal Delivery Systems and Topical Patches for ANDAs Draft Guidance for Industry.^20^ This 5-point score rates adhesion from 0 “≥ 90% adhered, essentially no lifting off the skin” to 4 “0% adhered, the patch/adhesive is detached and is completely off the skin”. The scale was used to assess the adhesion of the dressing at the end of infusion prior to SQIN-Infusor removal.
4. *Pharmacokinetics:* Plasma furosemide concentration was measured at 0 (pre-dose), 60 and 240 minutes after the start of SC furosemide infusion.

*Secondary outcomes:*

1. *Pharmacodynamics:* Urine volume and urine sodium concentration 8 hours after the start of SC furosemide infusion.
2. *Local skin reactions*: Assessment for skin irritation was completed using photography and a standardised scale to assess erythema and oedema formation as recommended by the National Academy of Sciences. This 6-point scale ranges from 0 representing “no response” to 4 representing “bullous (large blister), spreading, or other severe reaction”.^19^ Skin appearance was assessed at following timepoints: pre-dose, after SQIN-Infusor removal and 24 hours after the start of infusion.
3. *Patient acceptability*: The usability of the SQIN-Infusor was assessed using SUS. SUS is an instrument commonly used to test usability in commercial products.^4,21^ It consists of ten questions with five-point response options from 1 (strongly disagree) to 5 (strongly agree). It provides a score from 0 to 100, with scores >85 representing exceptional usability and a score <70 representing unacceptable usability.^4^ The survey was completed by all participants following the end of treatment.

**Blood sample analysis**

Similarly to SQIN-Furosemide PK/PD study, all samples were collected at prespecified nominal timepoints, processed and stored at -20^o^ C or colder until assayed using the same liquid chromatography tandem-mass spectrometry method.

**Statistical analysis**

The analysis population included all participants in whom SQIN-Infusor was activated.

All primary and secondary safety outcomes were listed by participant or summarised using descriptive statistics, as appropriate. AEs and device failure were summarised for each event. Plasma furosemide concentrations at pre-specified times (0, 60 and 240 minutes from start of the infusion) were summarised using descriptive statistics (including N, mean, standard deviation, coefficient of variation, median, minimum and maximum). Urine volume and spot sodium concentration measured at 8 hours were summarised using descriptive statistics (including N, mean, standard deviation, coefficient of variation, median, minimum and maximum).
